# Redox regulation of responses to hypoxia and NO-cGMP signaling in pulmonary vascular pathophysiology

**DOI:** 10.1186/1471-2210-11-S1-O8

**Published:** 2011-08-01

**Authors:** Michael S Wolin, Boon Hwa Neo, Dhara Patel, Raed Alhawaj, Sharath Kandhi, Mansoor Ahmad

**Affiliations:** 1 Department of Physiology, New York Medical College, Valhalla, NY 10595, USA

## 

There is much controversy in mechanisms controlling the pulmonary arterial response to acute and chronic hypoxia. Our previous work suggests bovine pulmonary arteries (BPA) contract to hypoxia via removal of a relaxation mediated by hydrogen peroxide derived from superoxide generated by Nox4 [1]. In contrast, bovine coronary arteries (BCA) relax to hypoxia via cytosolic NADPH oxidation coordinating multiple processes that lower intracellular calcium [2]. Peroxide appears to relax BPA by both stimulation of soluble guanylate cyclase (sGC) and by a cGMP-independent activation of protein kinase G (PKG) resulting from thiol oxidation-mediated subunit dimerization [3]. A removal of both of these mechanisms appears to contribute to the hypoxic pulmonary vasoconstriction (HPV) response seen in BPA. In addition, PKG dimerization appears to participate in the relaxation of coronary arteries to hypoxia. BPA secrete superoxide derived from Nox2 into the extracellular environment, and increased extracellular SOD (ecSOD) activity appears to attenuate the HPV response by increasing extracellular peroxide levels from this extracellular source of superoxide. Increased peroxide generated by Nox2 or mitochondria also appears to attenuate the relaxation of BCA to hypoxia by stimulating ERK MAP kinase [4]. Exposure of mice to 21 days of hypoxia (10% O_2_) promotes pulmonary hypertension associated with decreased pulmonary artery and aortic contraction to phenylephrine, NO-mediated relaxation to acetylcholine (ACh) and responses to hypoxia. While increased pulmonary arterial expression of sGC has been reported in this mouse model of pulmonary hypertension, chronic hypoxia had minimal effects on relaxation to an NO donor. Catalase markedly restored contraction to phenylephrine and the aortic relaxation to hypoxia. Chronic treatment of mice with cobalt protoporphyrin IX, which induces heme oxygenase and ecSOD, attenuated the development of pulmonary hypertension without restoring relaxation to ACh. In addition, chronic treatment of mice with delta-aminolevulinic acid, a precursor to the sGC activator protoporphyrin IX and heme needed for sGC activation and heme oxygenase activity, also attenuated pulmonary hypertension development without restoring responses to ACh. Thus, redox mechanisms regulating PKG seem to be important contributors to vascular oxygen sensing mechanisms. In addition, increased ecSOD expression and PKG-mediated vasodilation to endogenously generated peroxide and other sGC activators may function as a protective mechanism against the development of hypoxia-induced pulmonary hypertension.
